# Mortality risk factors in primary Sjögren syndrome: a real-world, retrospective, cohort study

**DOI:** 10.1016/j.eclinm.2023.102062

**Published:** 2023-07-04

**Authors:** Pilar Brito-Zerón, Alejandra Flores-Chávez, Ildiko Fanny Horváth, Astrid Rasmussen, Xiaomei Li, Peter Olsson, Arjan Vissink, Roberta Priori, Berkan Armagan, Gabriela Hernandez-Molina, Sonja Praprotnik, Luca Quartuccio, Nevsun Inanç, Burcugül Özkızıltaş, Elena Bartoloni, Agata Sebastian, Vasco C. Romão, Roser Solans, Sandra G. Pasoto, Maureen Rischmueller, Carlos Galisteo, Yasunori Suzuki, Virginia Fernandes Moça Trevisani, Cecilia Fugmann, Andrés González-García, Francesco Carubbi, Ciprian Jurcut, Toshimasa Shimizu, Soledad Retamozo, Fabiola Atzeni, Benedikt Hofauer, Sheila Melchor-Díaz, Tamer Gheita, Miguel López-Dupla, Eva Fonseca-Aizpuru, Roberto Giacomelli, Marcos Vázquez, Sandra Consani, Miriam Akasbi, Hideki Nakamura, Antónia Szántó, A. Darise Farris, Li Wang, Thomas Mandl, Angelica Gattamelata, Levent Kilic, Katja Perdan Pirkmajer, Kerem Abacar, Abdurrahman Tufan, Salvatore de Vita, Hendrika Bootsma, Manuel Ramos-Casals, Sjögren Big Data Consortiumar, S. Arends, E. Treppo, S. Longhino, V. Manfrè, M. Rizzo, C. Baldini, S. Bombardieri, M. Bandeira, M. Silvéiro-António, R. Seror, X. Mariette, G. Nordmark, D. Danda, P. Wiland, R. Gerli, S.K. Kwok, S.H. Park, M. Kvarnstrom, M. Wahren-Herlenius, S. Downie-Doyle, D. Sene, D. Isenberg, V. Valim, V. Devauchelle-Pensec, A. Saraux, J. Morel, C. Morcillo, P.E. Díaz Cuiza, B.E. Herrera, L. González-de-Paz, A. Sisó-Almirall

**Affiliations:** a Autoimmune Diseases Unit, Research and Innovation Group in Autoimmune Diseases, Sanitas Digital Hospital, Hospital-CIMA-Centre Mèdic Milenium Balmes Sanitas, Barcelona, Spain; b Department of Autoimmune Diseases, ICMiD, Hospital Clínic, Barcelona, Spain; c Division of Clinical Immunology, Faculty of Medicine, University of Debrecen, Debrecen, Hungary; d Genes and Human Disease Research Program, Oklahoma Medical Research Foundation, Oklahoma City, OK, USA; e Department of Rheumatology and Immunology, The First Affiliated Hospital of USTC, University of Science and Technology of China(Anhui Provincial Hosipital), Hefei, China; f Department of Rheumatology, Skane University Hospital Malmö, Lund University, Lund, Sweden; g Department of Oral and Maxillofacial Surger, University of Groningen, University Medical Centre Groningen, Groningen, the Netherlands; h Department of Internal Medicine and Medical Specialties, Rheumatology Clinic, Sapienza University of Rome, Rome, Italy; i Saint Camillus International University of Health Science, UniCamillus, Rome, Italy; j Division of Rheumatology, Department of Internal Medicine, Hacettepe University, Faculty of Medicine, Ankara, Turkey; k Immunology and Rheumatology Department, Instituto Nacional de Ciencias Médicas y Nutrición Salvador Zubirán, México City, Mexico; l Department of Rheumatology, University Medical Centre, Ljubljana, Slovenia; m Clinic of Rheumatology, Department of Medical and Biological Sciences, University Hospital “Santa Maria della Misericordia”, Udine, Italy; n Marmara University, School of Medicine, Istanbul, Turkey; o Division of Rheumatology, Department of Internal Medicine, Gazi University School of Medicine, Ankara, Turkey; p Rheumatology Unit, Department of Medicine, University of Perugia, Perugia, Italy; q Department of Rheumatology and Internal Medicine, Wroclaw Medical Hospital, Wroclaw, Poland; r Rheumatology Department, Hospital de Santa Maria, Centro Hospitalar Universitário Lisboa Norte and Rheumatology Research Unit, Instituto de Medicina Molecular, Faculdade de Medicina, Universidade de Lisboa, Lisbon Academic Medical Centre, Lisbon, Portugal; s Department of Internal Medicine, Hospital Vall d'Hebron, Barcelona, Spain; t Rheumatology Division, Hospital das Clinicas HCFMUSP, Faculdade de Medicina da Universidade de Sao Paulo, Sao Paulo, Brazil; u Department of Rheumatology, The Queen Elizabeth Hospital, Discipline of Medicine, University of Adelaide, South Australia, Australia; v Department of Rheumatology, Hospital Parc Taulí, Barcelona, Spain; w Division of Rheumatology, Kanazawa University Hospital, Kanazawa, Ishikawa, Japan; x Division of Health Based Evidence, Federal University of São Paulo, Sao Paulo, Brazil; y Rheumatology, Department of Medical Sciences, Uppsala University, Uppsala, Sweden; z Department of Rheumatology, Hospital 12 de Octubre, Madrid, Spain; aa Internal Medicine and Nephrology Unit, Department of Medicine, ASL Avezzano-Sulmona-L'Aquila, San Salvatore Hospital, L'Aquila, Italy; ab Department of Internal Medicine, Carol Davila Central Military Emergency Hospital, Bucharest, Romania; ac Division of Advanced Preventive Medical Sciences, Department of Immunology and Rheumatology, Nagasaki University Graduate School of Biomedical Sciences, Nagasaki, Japan; ad Department of Rheumatology, Hospital Quirón Salud, Barcelona, Spain; ae IRCCS Galeazzi Orthopedic Institute, Milan and Rheumatology Unit, University of Messina, Messina, Italy; af Otorhinolaryngology, Head and Neck Surgery, Technical University Munich, Munich, Germany; ag Department of Rheumatology, Hospital 12 de Octubre, Madrid, Spain; ah Rheumatology Department, Kasr Al Ainy School of Medicine, Cairo University, Cairo, Egypt; ai Department of Internal Medicine, Hospital Joan XXIII, Tarragona, Spain; aj Department of Internal Medicine, Hospital de Cabueñes, Gijón, Spain; ak Clinical Unit of Rheumatology, University of l'Aquila, School of Medicine, L'Aquila, Italy; al Department of Rheumatology, Hospital de Clínicas, San Lorenzo, Paraguay; am Internal Medicine, Hospital Maciel, and Universidad de la República (UdelaR), Montevideo, Uruguay; an Department of Internal Medicine, Hospital Infanta Leonor, Madrid, Spain; ao Division of Hematology and Rheumatology, Department of Medicine, Nihon University School of Medicine, Oyaguchi Kami-cho, Itabashi-ku, Tokyo, Japan; ap Department of Rheumatology & Clinical Immunology, University of Groningen, University Medical Centre Groningen, Groningen, the Netherlands; aq Department of Medicine, University of Barcelona, Barcelona, Spain

**Keywords:** Sjögren syndrome, Mortality, Systemic disease, Lymphoma, Cardiovascular, Infection

## Abstract

**Background:**

What baseline predictors would be involved in mortality in people with primary Sjögren syndrome (SjS) remains uncertain. This study aimed to investigate the baseline characteristics collected at the time of diagnosis of SjS associated with mortality and to identify mortality risk factors for all-cause death and deaths related to systemic SjS activity measured by the ESSDAI score.

**Methods:**

In this international, real-world, retrospective, cohort study, we retrospectively collected data from 27 countries on mortality and causes of death from the Big Data Sjögren Registry. Inclusion criteria consisted of fulfilling 2002/2016 SjS classification criteria, and exclusion criteria included chronic HCV/HIV infections and associated systemic autoimmune diseases. A statistical approach based on a directed acyclic graph was used, with all-cause and Sjögren-related mortality as primary endpoints. The key determinants that defined the disease phenotype at diagnosis (glandular, systemic, and immunological) were analysed as independent variables.

**Findings:**

Between January 1st, 2014 and December 31, 2023, data from 11,372 patients with primary SjS (93.5% women, 78.4% classified as White, mean age at diagnosis of 51.1 years) included in the Registry were analysed. 876 (7.7%) deaths were recorded after a mean follow-up of 8.6 years (SD 7.12). Univariate analysis of prognostic factors for all-cause death identified eight Sjögren-related variables (ocular and oral tests, salivary biopsy, ESSDAI, ANA, anti-Ro, anti-La, and cryoglobulins). The multivariate CPH model adjusted for these variables and the epidemiological features showed that DAS-ESSDAI (high vs no high: HR = 1.68; 95% CI, 1.27–2.22) and cryoglobulins (positive vs negative: HR = 1.72; 95% CI, 1.22–2.42) were independent predictors of all-cause death. Of the 640 deaths with available information detailing the specific cause of death, 14% were due to systemic SjS. Univariate analysis of prognostic factors for Sjögren-cause death identified five Sjögren-related variables (oral tests, clinESSDAI, DAS-ESSDAI, ANA, and cryoglobulins). The multivariate competing risks CPH model adjusted for these variables and the epidemiological features showed that oral tests (abnormal vs normal results: HR = 1.38; 95% CI, 1.01–1.87), DAS-ESSDAI (high vs no high: HR = 1.55; 95% CI, 1.22–1.96) and cryoglobulins (positive vs negative: HR = 1.52; 95% CI, 1.16–2) were independent predictors of SjS-related death.

**Interpretation:**

The key mortality risk factors at the time of SjS diagnosis were positive cryoglobulins and a high systemic activity scored using the ESSDAI, conferring a 2-times increased risk of all-cause and SjS-related death. ESSDAI measurement and cryoglobulin testing should be considered mandatory when an individual is diagnosed with SjS.

**Funding:**

10.13039/100004336Novartis.

**Research in context**

**Evidence before this study**

Sjögren syndrome (SjS) is a systemic autoimmune disease for which it remains uncertain whether it has a reduced life expectancy. We searched PubMed for studies published from 1998 to January 31, 2023, with the terms “Sjögren syndrome” AND “mortality risk factors”. We also included publications cited in the documents when relevant. We did not use any restrictions for the language of the published studies. The criteria used to include or exclude studies consisted of the fulfilment of the current classification criteria (2002, 2016, or equivalent), and a statistical approach mainly based on Cox-Proportional Hazard models.

These studies have been centered on analysing the predictors of all-cause and SjS-related causes of death in populations of less than 1000 people, yielding that the number of events of interest (all-cause deaths) is too low, ranging between 30 and 50. These small samples limit the reliability of identifying mortality risk factors and prevent detailed analysis of different causes of death when stratified. Additionally, all these studies, except for one, did not classify systemic activity using the ESSDAI definitions and scoring and did not study predictors for specific causes of death.

**Added value of this study**

The study identified all-cause and SjS-related death predictors in the largest cohort (12,000 patients) with the broadest international representation (27 countries from 5 continents). Baseline predictors included positive cryoglobulins (identified as an independent variable in the five multivariate-adjusted CPH models), high systemic activity (identified in three models), and abnormal results in oral tests (identified in two models), conferring an increased risk of death of 1.7–1.9 for all-cause death and 1.4–2.6 for SjS-related death. Although it was not the objective of this study, high systemic activity at diagnosis was also related to poor survival due to infections and cardiovascular disease.

**Implications of all the available evidence**

The findings support the mandatory measurement at SjS diagnosis of systemic activity using the ESSDAI score and cryoglobulin testing to anticipate better which patients may have poor outcomes and will require closer follow-up.

## Introduction

Sjögren syndrome (SjS) is a systemic autoimmune disease that primarily affects women between the fourth and sixth decades of life, but it also can be diagnosed at any age.^1^ SjS is often considered a chronic, non-life-threatening disease. Dryness is the key clinical feature of SjS, affecting more than 95% of patients.^1^ It has a strong relationship with physical fatigue, pain, and depression,^2^ conforming to a conundrum of symptoms that are not life-threatening but dramatically influence the quality of life. However, the clinical spectrum of SjS can extend from these symptoms to a range of systemic organ-specific manifestations that may present at diagnosis or develop later, clearly influencing the prognosis of the disease.^3^^,^^4^ Systemic involvement was inadequately recognized as a crucial part of the disease phenotype until 2010, when the EULAR SjS disease activity index (ESSDAI) was published. The ESSDAI is now the standard tool for evaluating systemic activity in individuals with SjS.^5^

What baseline predictors would be involved in mortality in people with SjS remains uncertain.^6^ Several studies have measured the standardized mortality rate (SMR) to evaluate whether individuals with SjS are more, less, or equally likely to die compared to the general population. These studies have yielded inconsistent findings, including some showing no significant differences in mortality (SMRs ranging from 1.15 to 1.47)^7^, ^8^, ^9^, ^10^, ^11^ and others indicating higher mortality rates in SjS (SMRs ranging from 2.07 to 4.66).^6^^,^^12^^,^^13^ Other research has sought to analyse the causes of death and mortality risk factors.^6^^,^^10^^,^^14^, ^15^, ^16^ However, these studies often included less than 1000 individuals with SjS and reported mortality rates below 10%, resulting in sample sizes ranging from 30 to 50 among those who died. These small samples limit the reliability of identifying mortality risk factors and prevent detailed analysis of different causes of death when stratified. Additionally, all these studies except for one^17^ did not classify systemic activity using the ESSDAI definitions and scoring.

The Sjögren Big Data Consortium^18^ is the largest real-world international SjS registry, including over 10,000 well-characterized individuals in which systemic activity is collected using the ESSDAI score. We conducted an exploratory study to identify the baseline characteristics of SjS collected at the time of diagnosis associated with mortality. We searched for mortality risk factors for all-cause death and deaths related to systemic SjS activity as measured by the ESSDAI.

## Methods

### Patients

The Sjögren Big Data Consortium is an international, multicentre registry established in 2014 to provide a real-world understanding of the phenotypes and outcomes of individuals with primary SjS through the cooperative merging of existing individual databases from leading centres in SjS clinical research. Inclusion criteria for the registry consisted of a clinical diagnosis of primary SjS based on fulfilling 2002/2016 classification criteria.^19^^,^^20^ Exclusion criteria for considering SjS as a primary disease were chronic HCV/HIV infections and associated systemic autoimmune diseases (at diagnosis or during the follow-up). The longitudinal cohort collected cases diagnosed before 2014 and incident cases diagnosed after 2014 (individual databases are updated annually).

The database consisted of a minimum basic data set (MBDS) of variables considered essential for characterizing the disease phenotype at the time of diagnosis: age, sex, ethnicity, country of residence, ocular and oral dryness, ocular and oral tests, salivary gland biopsy, global clinical ESSDAI scores (clinESSDAI), antinuclear antibodies, rheumatoid factor, anti-Ro and anti-La antibodies, C3 and C4 levels, and cryoglobulins. Diagnostic tests for SjS (ocular tests, oral tests, and salivary gland biopsy) were conducted according to the recommendations of 2002/2016 criteria.^19^^,^^20^ The attending physician verified causes of death according to the information from electronic healthcare databases and national death databases and classified them into the following categories: Sjögren-related (the leading cause of death was related to systemic SjS involvement defined according to the ESSDAI), infection, cardiovascular disease, solid neoplasia, and other causes.^17^^,^^21^

To harmonize databases from each centre, we applied specific pre-processing techniques such as detecting and treating outliers, influential observations, errors in naming standards, and other errors, and excluding observations with missing data for mandatory variables (age, sex, symptoms, criteria fulfillment). The study was approved by the Ethics Committee of the coordinating centre, and live participants provided written/oral informed consent. (Hospital Clinic, Barcelona, Spain, registry HCB/2015/0869). The study adhered to the STROBE reporting guidelines.

### Dependent and independent variables

**Fig. 1:**
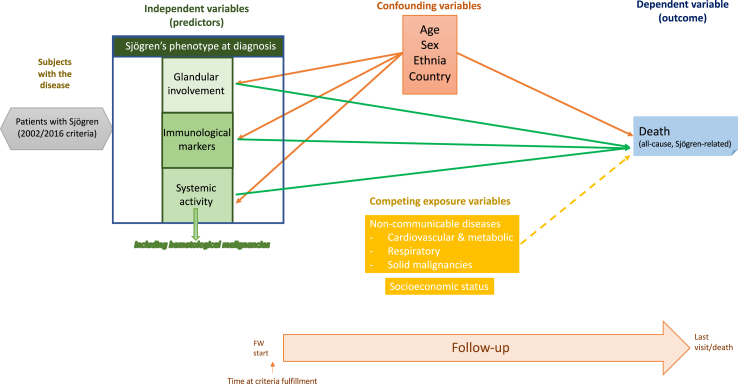
Design of the statistical approach of the study based on a directed acyclic graph including all-cause and SjS-related mortality as dependent variables, and the following variables classified as: • Independent variables (those that define the disease phenotype at the time of diagnosis, grouped into three categories: glandular involvement, immunological variables, and systemic involvement). They are the variables that characterize the disease at the time of its diagnosis and that have been related to the prognosis of the disease according to previous studies. • Confounding variables: the epidemiological variables that have been related to both the independent variables (phenotype of the disease) and the dependent variable to be studied (mortality) according to previous studies. • Competing exposure variables (socioeconomic status, non-communicable diseases): variables that can influence the dependent variable to study but for which no published evidence supports a significant influence in the disease phenotype (independent variables).

A statistical approach was designed using a directed acyclic graph (DAG) (Fig. 1). The primary endpoints (dependent variables) were all-cause and Sjögren-related mortality. The key determinants that defined the disease phenotype at the time of diagnosis were grouped into three subsets (glandular, systemic, and immunological) and were classified as independent variables due to their potential impact on poor outcomes based on previous research^1^^,^^22^; a previous history of haematological malignancy was also included in the adjusted models. Among covariates, epidemiological features (age at diagnosis, sex, ethnicity following the FDA recommendations,^17^^,^^21^ and WHO country-related universal health coverage -UHC- score) were analysed as confounding variables due to their influence in both the independent variables (disease phenotype at diagnosis) and the outcome measured (death).

### Statistical analysis

Descriptive data are presented as mean and standard deviation (SD) for continuous variables and numbers and percentages (%) for categorical variables. The effect of phenotypic determinants at diagnosis on mortality was assessed using univariate Cox proportional hazards (CPH) regression analysis. Variables corresponding to disease determinants were dichotomized; the value considered as a prognostic factor was defined according to the results obtained in previous studies.^1^^,^^22^ SjS individuals were stratified according to the presence or absence of each prognostic factor at the time of diagnosis. A multivariate-adjusted CPH model was designed to identify prognostic factors for death (all-cause and Sjögren-related cause), including the analysis of the proportional assumption of the model. The fully adjusted model included the epidemiological features and the phenotypic variables identified in the univariate analysis with a P value < 0.1. The hazard ratios (HRs) and their 95% confidence intervals (CIs) obtained in the CPH proportional-hazards regression analysis were calculated.

The start of the follow-up time for each patient was defined according to the date when the physician responsible for the patient's follow-up confirmed criteria fulfillment. The end of the follow-up time for each patient was defined according to the last recorded National Healthcare System visit. Patients with less than six months of follow-up after criteria fulfillment were excluded. We restricted the Kaplan–Meier analysis to 20-year follow-up. The Log Rank-Mantel-Cox test was used to compare the survival curves between patients with and without the determinants. All studies were conducted using the R software package (R version 4.2.3).

We carried out the following post-hoc sensitivity analyses: 1) Stratification of systemic activity according to the systemic disease activity states (DAS) using a univariate CPH model: no activity (global score = 0), low activity (global score 1–4), moderate activity (global score 5–13) and high activity (global score ≥14)^23^; 2) Stratification of systemic activity according to the number of clinESSDAI domains that were scored as having the highest level of activity at the time of diagnosis (none, one, or two or more clinical domains scored as high activity)^24^ using a univariate CPH model; 3) Inclusion of people followed lower than six months; and 4) Competing risks regression model to analyse the potential influence of causes of death unrelated to SjS (competing events) in the risk of death from systemic SjS (event at interest), using a CPH regression model treating competing events as right-censored observations.

### Role of the funding source

The funder of the study had no role in study design, data collection, data analysis, data interpretation, or writing of the report. MRC, PBZ & AFC has directly accessed and verified the merged data. MRC was responsible for the decision to submit the manuscript.

## Results

Among the 15,654 patients available in the Big Data Registry by April 2022, 2852 cases were excluded from the survival analysis due to a lack of follow-up, and 1430 due to a follow-up period of fewer than six months. Therefore, we analysed the data from 11,372 cases (93.5% women, 78.4% classified as White, with a mean age at diagnosis of 51.1 years, SD 14.4); 229 (2%) fulfilled the 2002 classification criteria only (positive anti-La in the absence of anti-Ro antibodies). Baseline features are summarized in Table 1.

**Table 1:** Baseline features of 11,372 patients at diagnosis of primary SjS.

Epidemiological determinants	Variables	N = 11,372	%
Epidemiological	Sex (female)	10,629	93.5
	Mean age (+SD)	51.11 (14.45)	
	Ethnicity		
	White	8901	78.27
	Asian	1528	13.44
	Hispanic	777	6.83
	*Black-African American (BAA)*	134	1.18
	*Others*	32	0.28
SjS-related determinants			
Glandular involvement	Dryness of the mouth and/or eyes	10,973	96.5
	*Dryness of the mouth*	10,350	91.01
	*Dryness of the eyes*	10,291	90.49
	Ocular tests (abnormal results)	8677/10,136	85.6
	Oral tests (abnormal results)	6121/7647	80
	Focal lymphocytic sialadenitis	6677/7695	86.8
Systemic activity	Systemic activity (clinESSDAI >0)	7268	63.9
	DAS		
	*No activity*	4104	36.1
	*Low activity*	2520	22.2
	*Moderate activity*	3289	28.9
	*High activity*	1459	12.8
Immunological markers	Antinuclear antibodies	9272/11,148	83.2
	Rheumatoid factor	4895/10,455	46.8
	Ro autoantibodies	8534/11,273	75.7
	La autoantibodies	4815/11,181	43.1
	Low C3 value	1386/9986	13.9
	Low C4 value	1151/9952	11.6
	Serum cryoglobulins	484/5678	8.5

### All-cause death

Patients were followed for a mean of 8.6 years (SD 7.12 years), and 876 (7.7%) deaths were recorded. The overall cumulative risk of all-cause death in the entire cohort was 4.95% (95% confidence interval [CI], 4.94%–4.96%) at 5 years, 9.74% (95% CI, 9.72–9.77) at 10 years, 18.50% (95% CI, 18.39–18.60%) at 20 years, and 25.69% (95% CI, 25.42–25.97%) at 30 years, respectively.

Univariate analysis of prognostic factors for all-cause death identified eight Sjögren-related variables (ocular tests, oral tests, SGB, DAS-ESSDAI, ANA, anti-Ro antibodies, anti-La antibodies, and cryoglobulins) with a P-value <0.1 (Table 2). In the multivariate-adjusted CPH model, DAS-ESSDAI (high vs no high: HR = 1.68; 95% CI, 1.27–2.22) and cryoglobulins (positive vs negative: HR = 1.72; 95% CI, 1.22–2.42) were independent predictors of all-cause death (Table 3). Sensitivity analysis including people followed for less than six months yielded similar HRs, identifying oral tests as an additional independent predictor for all-cause death (abnormal vs normal results: HR = 1.94; 95% CI, 1.17–3.23) (Supplementary Tables S1 and S2).

**Table 2:** Predictive factors for all-cause death identified in the univariate CPH model.

Domains	Variables at diagnosis	Reference	Group at risk	Univariate model for all-cause death				
				Coefficient (B)	HR	95% CI (I)	95% CI (S)	P-value
Epidemiological	Age	–	–	0.113	1.120	1.112	1.127	**<0.001**
	Sex	Women	Men	0.576	1.736	1.377	2.118	**<0.001**
	Ethnicity	White	Non-White	0.156	1.167	0.945	1.445	0.149
	Healthcare score^a^	>80	<80	−0.243	0.784	0.668	0.921	**0.003**
	History of HM	No	Yes	0.940	2.561	1.660	3.949	**<0.001**
Glandular	Dryness of mouth/eyes	Absence	Presence	−0.125	0.778	0.499	1.213	0.268
	Ocular tests	Normal	Abnormal	0.529	1.698	1.291	2.235	**<0.001**
	Oral tests	Normal	Abnormal	0.542	1.719	1.335	2.213	**<0.001**
	Salivary gland biopsy	Normal	FLS	−0.265	0.767	0.603	0.976	**0.031**
Systemic	clinESSDAI score	0	≥1	−0.011	0.989	0.880	1.161	0.875
	DAS state	No high	High	0.724	2.063	1.768	2.407	**<0.001**
Immunological	ANA	Negative	Positive	−0.152	0.858	0.721	1.021	**0.085**
	RF	Negative	Positive	−0.042	0.960	0.836	1.101	0.551
	Ro antibodies	Negative	Positive	−0.315	0.730	0.631	0.844	**<0.001**
	La antibodies	Negative	Positive	−0.131	0.877	0.767	1.002	**0.054**
	C3 levels	Normal	Low	−0.071	0.932	0.757	1.148	0.507
	C4 levels	Normal	Low	0.059	1.061	0.853	1.319	0.597
	Cryoglobulins	Negative	Positive	0.330	1.391	1.095	1.768	**0.007**

HM: Hematological malignancy; ANA: Antinuclear antibodies; RF: Rheumatoid factor; FLS: Focal lymphocytic sialoadenitis; DAS: Disease activity state; HR: Hazard ratio; CI: Confidence interval.

P value < 0.1 are indicated in bold.

a WHO-UHC score country by country.

**Table 3:** Predictive factors for all-cause death identified in the multivariate CPH model.

Domains	Variables at diagnosis	Reference	Group at risk	Multivariate adjusted model for all-cause death				
				Coefficient (B)	HR	95% CI (I)	95% CI (S)	P-value
Epidemiological	Age	–	–	0.125	**1.133**	**1.118**	**1.149**	<0.001
	Sex	Women	Men	0.470	1.600	0.991	2.582	0.054
	Ethnicity	White	Non-White	−0.421	0.656	0.343	1.257	0.204
	Healthcare score^a^	>80	<80	0.072	1.075	0.788	1.466	0.648
	History of HM	No	Yes	−0.565	0.569	0.224	1.446	0.236
Glandular	Ocular tests	Normal	Abnormal	0.263	1.300	0.736	2.297	0.366
	Oral tests	Normal	Abnormal	0.438	1.550	0.986	2.437	0.057
	Salivary gland biopsy	Normal	FLS	−0.221	0.802	0.564	1.141	0.220
Systemic	DAS state	No high	High	0.520	**1.682**	**1.273**	**2.223**	<0.001
Immunological	ANA	Negative	Positive	0.169	1.184	0.834	1.681	0.344
	Ro antibodies	Negative	Positive	0.008	1.008	0.752	1.353	0.956
	La antibodies	Negative	Positive	−0.072	0.931	0.695	1.246	0.630
	Cryoglobulins	Negative	Positive	0.543	**1.721**	**1.222**	**2.423**	0.002

HM: Hematological malignancy; ANA: Antinuclear antibodies; FLS: Focal lymphocytic sialoadenitis; DAS: Disease activity state; HR: Hazard ratio; CI: Confidence interval.

The multivariate model included all the epidemiological variables as covariates and those that achieved a P-value <0.1 in the univariate analysis of Table 1 as independent variables. The global proportional assumption of the CPH model yielded a P-value of 0.37. P value < 0.1 are indicated in bold.

a WHO-UHC score country by country.

### Sjögren-related death

Of the 640 deaths with available information detailing the specific cause of death, 14% were due to systemic SjS (0.8% at any follow-up time); other causes of death included infection (28%), cardiovascular diseases (27%), solid malignancies (18%) and other (14%) (Supplementary Table S3).

Univariate analysis of prognostic factors for Sjögren-cause death identified five Sjögren-related variables (oral tests, clinESSDAI, DAS-ESSDAI, ANA, and cryoglobulins) with a P-value <0.1 (Table 4). In the multivariate-adjusted CPH model, cryoglobulins (positive vs negative: HR = 2.57; 95% CI, 1.28–5.17) were independent risk factors for Sjögren-related death (Table 5). Sensitivity analysis including people followed for less than six months yielded similar results (Supplementary Tables S4 and S5).

**Table 4:** Predictive factors for SjS-related death identified in the univariate CPH model.

Domains	Variables at diagnosis	Reference	Group at risk	Univariate model for SjS-related death				
				Coefficient (B)	HR	95% CI (I)	95% CI (S)	P-value
Epidemiological	Age	–	–	0.100	1.106	1.084	1.129	**<0.001**
	Sex	Women	Men	0.688	1.990	0.997	3.973	**0.051**
	Ethnicity	White	Non-White	0.008	1.008	0.556	1.828	0.978
	Healthcare score^a^	>80	<80	−1.192	0.304	0.157	0.588	**<0.001**
	History of HM	No	Yes	8.836	8.836	4.075	19.160	**<0.001**
Glandular	Dryness of mouth/eyes	Absence	Presence	0.585	1.795	0.249	12.922	0.561
	Ocular tests	Normal	Abnormal	0.452	1.572	0.724	3.412	0.253
	Oral tests	Normal	Abnormal	1.019	2.771	1.115	6.888	**0.028**
	Salivary gland biopsy	Normal	FLS	−0.090	0.913	0.388	2.151	0.836
Systemic	clinESSDAI score	0	≥1	0.816	2.263	1.332	3.844	**0.003**
	DAS state	No high	High	1.440	4.224	2.756	6.475	**<0.001**
Immunological	ANA	Negative	Positive	1.115	3.173	1.287	7.827	**0.012**
	RF	Negative	Positive	−0.011	0.989	0.637	1.534	0.959
	Ro antibodies	Negative	Positive	−0.106	0.899	0.555	1.458	0.667
	La antibodies	Negative	Positive	0.019	1.019	0.671	1.546	0.928
	C3 levels	Normal	Low	0.334	1.397	0.794	2.456	0.246
	C4 levels	Normal	Low	0.471	1.601	0.878	2.919	0.124
	Cryoglobulins	Negative	Positive	1.283	3.609	1.989	6.544	**<0.001**

HM: Hematological malignancy; ANA: Antinuclear antibodies; RF: Rheumatoid factor; FLS: Focal lymphocytic sialoadenitis; DAS: Disease activity state; HR: Hazard ratio; CI: Confidence interval.

P value < 0.1 are indicated in bold.

a WHO-UHC score country by country.

**Table 5:** Predictive factors for SjS-related death identified in the multivariate CPH model.

Domains	Variables at diagnosis	Reference	Group at risk	Multivariate adjusted model for SjS-related death				
				Coefficient (B)	HR	95% CI (I)	95% CI (S)	P-value
Epidemiological^b^	Age	–	–	0.129	**1.138**	**1.101**	**1.175**	<0.001
	Sex	Women	Men	0.878	2.406	0.994	5.828	0.052
	Healthcare score^a^	>80	<80	−0.855	0.425	0.167	1.082	0.073
	History of HM	No	Yes	0.938	2.555	0.822	7.942	0.105
Glandular	Oral tests	Normal	Abnormal	1.349	3.854	0.923	16.091	0.064
Systemic	clinESSDAI score	0	≥1	0.591	1.805	0.755	4.317	0.184
	DAS state	No high	High	0.553	1.739	0.845	3.579	0.133
Immunological	ANA	Negative	Positive	0.688	1.990	0.592	6.688	0.266
	Cryoglobulins	Negative	Presence	0.943	**2.568**	**1.277**	**5.166**	0.008

HM: Hematological malignancy; ANA: Antinuclear antibodies; RF: Rheumatoid factor; FLS: Focal lymphocytic sialoadenitis; DAS: Disease activity state; HR: Hazard ratio; CI: Confidence interval.

The multivariate model included all the epidemiological variables as covariates and those that achieved a P-value <0.1 in the univariate analysis of Table 3 as independent variables. The global proportional assumption of the CPH model yielded a P-value of 0.145. P value < 0.1 are indicated in bold.

a WHO-UHC score country by country.

b Ethnicity excluded from the model due to low number of events.

**Fig. 2:**
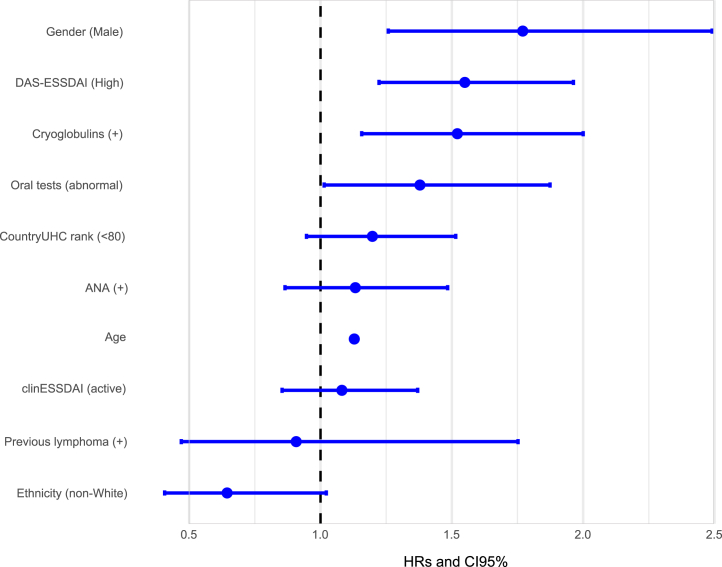
Forest plot of HRs and 95% confidence interval for SjS-related death yielded by the CPH regression model treating cases of death unrelated to Sjögren as competing events (right-censored observations).

**Fig. 3:**
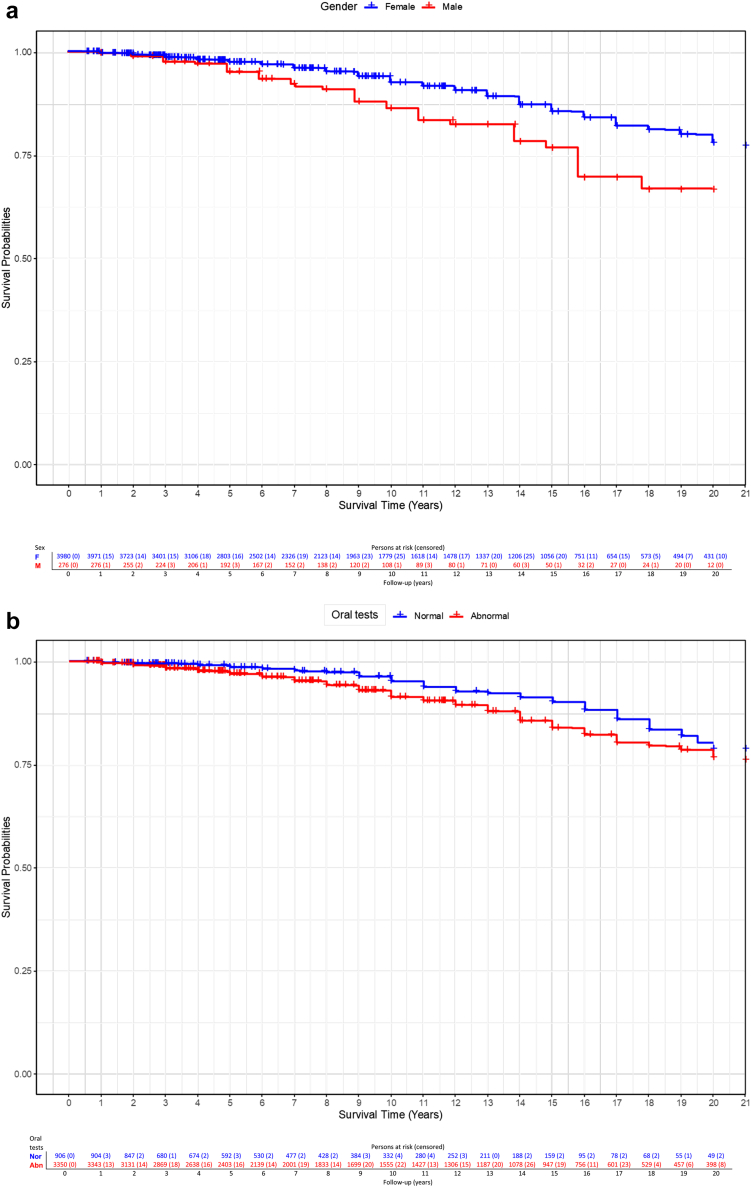

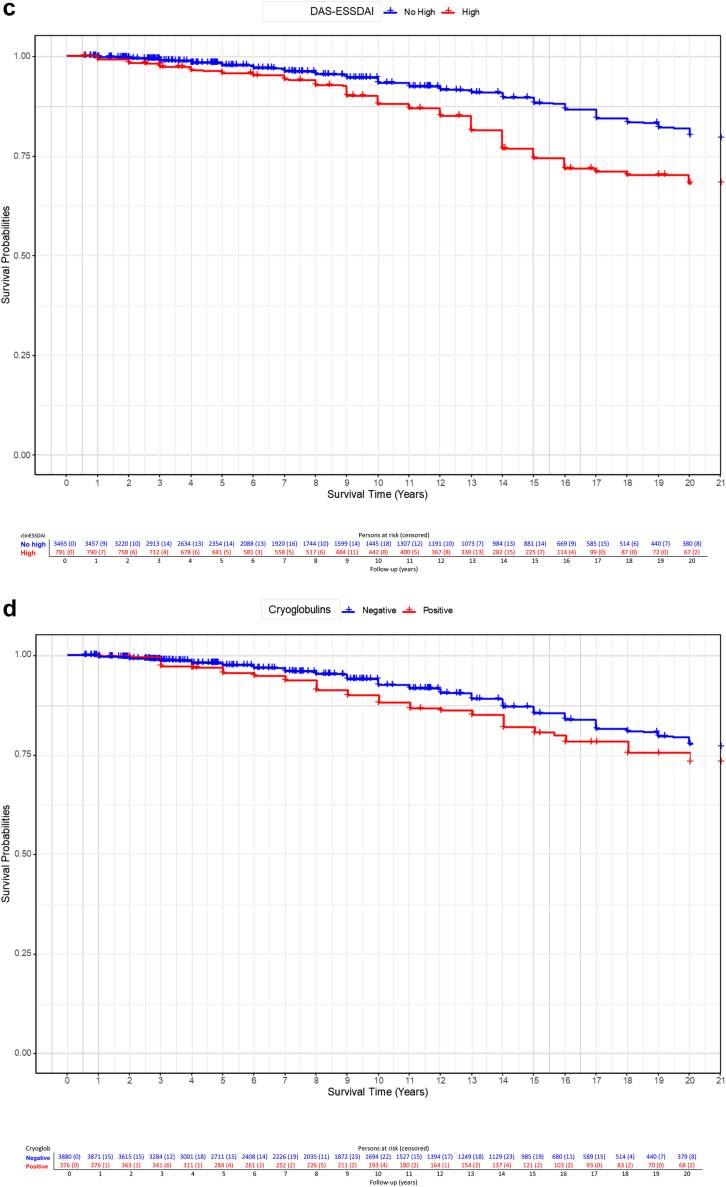
Kaplan–Meier analysis and Log-rank test comparing the survival curves for SjS related-deaths (treating other causes of death as competing events) during the first 20 years of follow-up of patients: 3a) males vs females; 3b) abnormal vs normal results in oral tests at baseline; 3c) high vs non-high DAS-ESSDAI at baseline; 3d) positive vs negative cryoglobulins at baseline.

In the multivariate-adjusted, competing risks CPH regression model, oral tests (abnormal vs normal results: HR = 1.38; 95% CI, 1.01–1.87), DAS-ESSDAI (high vs no high: HR = 1.55; 95% CI, 1.22–1.96) and cryoglobulins (positive vs negative: HR = 1.52; 95% CI, 1.16–2) were independent predictors of SjS-related death (Table 6, Fig. 2). Male patients had a decreased survival due to SjS-related death compared to females (Log Rank-Mantel-Cox test = 0.002, Fig. 3a), as well as those with abnormal compared to those with normal oral tests at the time of diagnosis (Log Rank-Mantel-Cox test = 0.002, Fig. 3b), those with high DAS-ESSDAI (Log Rank-Mantel-Cox test <0.001, Fig. 3c) and those with cryoglobulinaemia (Log Rank-Mantel-Cox test 0.046, Fig. 3d).

**Table 6:** Predictive factors for SjS-related death identified in a competing risk analysis with a multivariate CPH model in which causes of death unrelated to Sjögren were considered as right-censored competing events.

Domains	Variables at diagnosis	Reference	Group at risk	Multivariate adjusted model for SjS-related death (competing events)				
				Coefficient (B)	HR	95% CI (I)	95% CI (S)	P-value
Epidemiological	Age	–	–	0.121	**1.129**	**1.117**	**1.140**	<0.001
	Sex	Women	Men	0.571	**1.770**	**1.258**	**2.491**	0.001
	Ethnicity	White	Non-White	−0.439	0.644	0.407	1.022	0.062
	Healthcare score^a^	>80	<80	0.180	1.198	0.947	1.515	0.133
	History of HM	No	Yes	−0.097	0.908	0.470	1.752	0.773
Glandular	Oral tests	Normal	Abnormal	0.321	**1.379**	**1.014**	**1.874**	0.040
Systemic	clinESSDAI score	0	≥1	0.078	1.081	0.854	1.370	0.516
	DAS state	No high	High	0.438	**1.549**	**1.223**	**1.963**	<0.001
Immunological	ANA	Negative	Positive	0.125	1.133	0.865	1.484	0.365
	Cryoglobulins	Negative	Presence	0.419	**1.521**	**1.157**	**2.000**	0.003

HM: Hematological malignancy; ANA: Antinuclear antibodies; RF: Rheumatoid factor; FLS: Focal lymphocytic sialoadenitis; DAS: Disease activity state; HR: Hazard ratio; CI: Confidence interval.

The multivariate model included all the epidemiological variables as covariates and those that achieved a P-value <0.1 in the univariate analysis of Table 3 as independent variables. The proportional assumption of the global CPH model yielded a P-value of 0.109 (one variable had a P-value near 0.05). P value < 0.1 are indicated in bold.

a WHO-UHC score country by country.

**Fig. 4:**
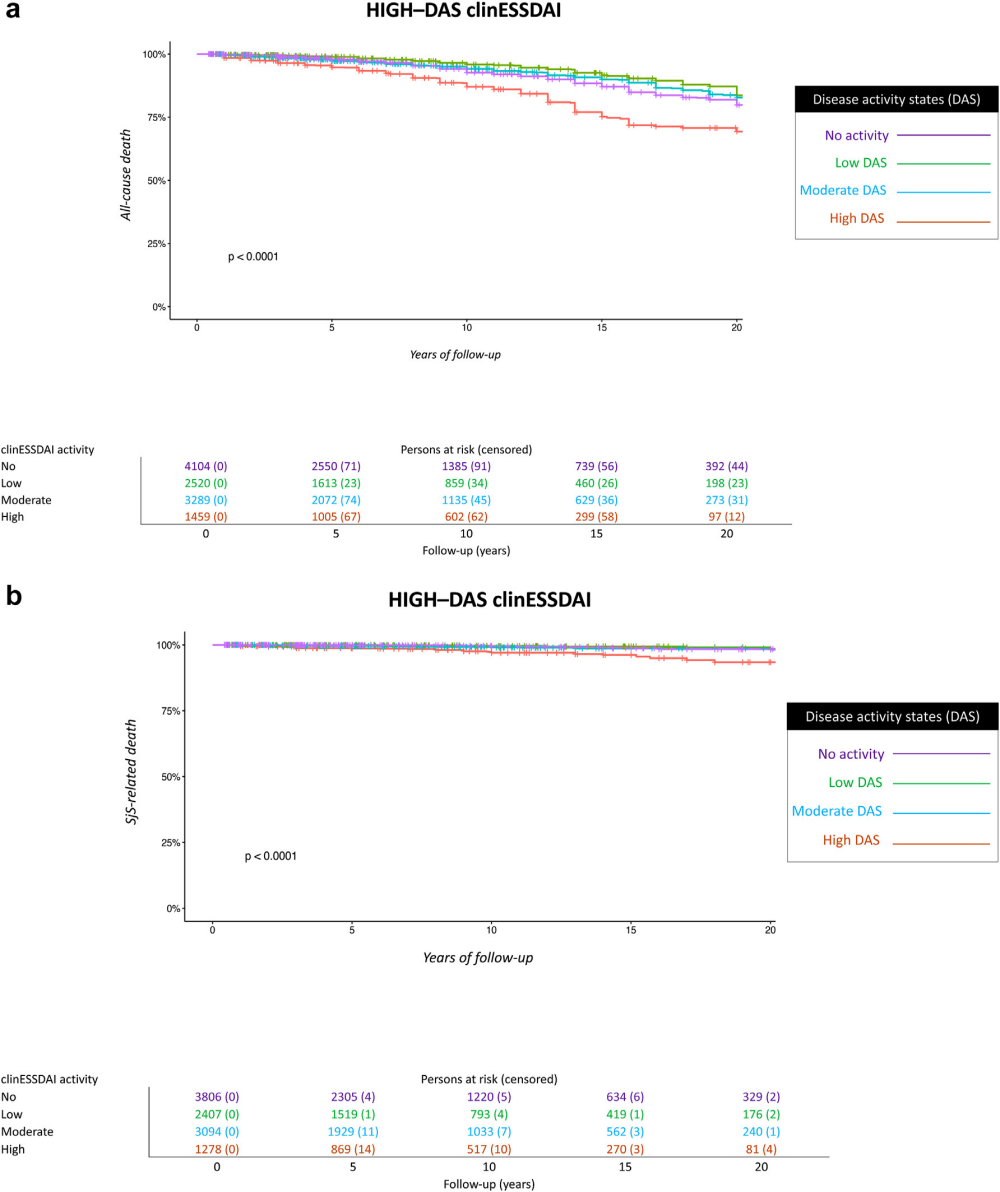
Cumulative survival for all-cause (**4a**) and SjS-related (**4b**) death according to the disease activity states (DAS) scored at the time of SjS diagnosis (no activity, low activity, moderate activity, high activity).

**Fig. 5:**
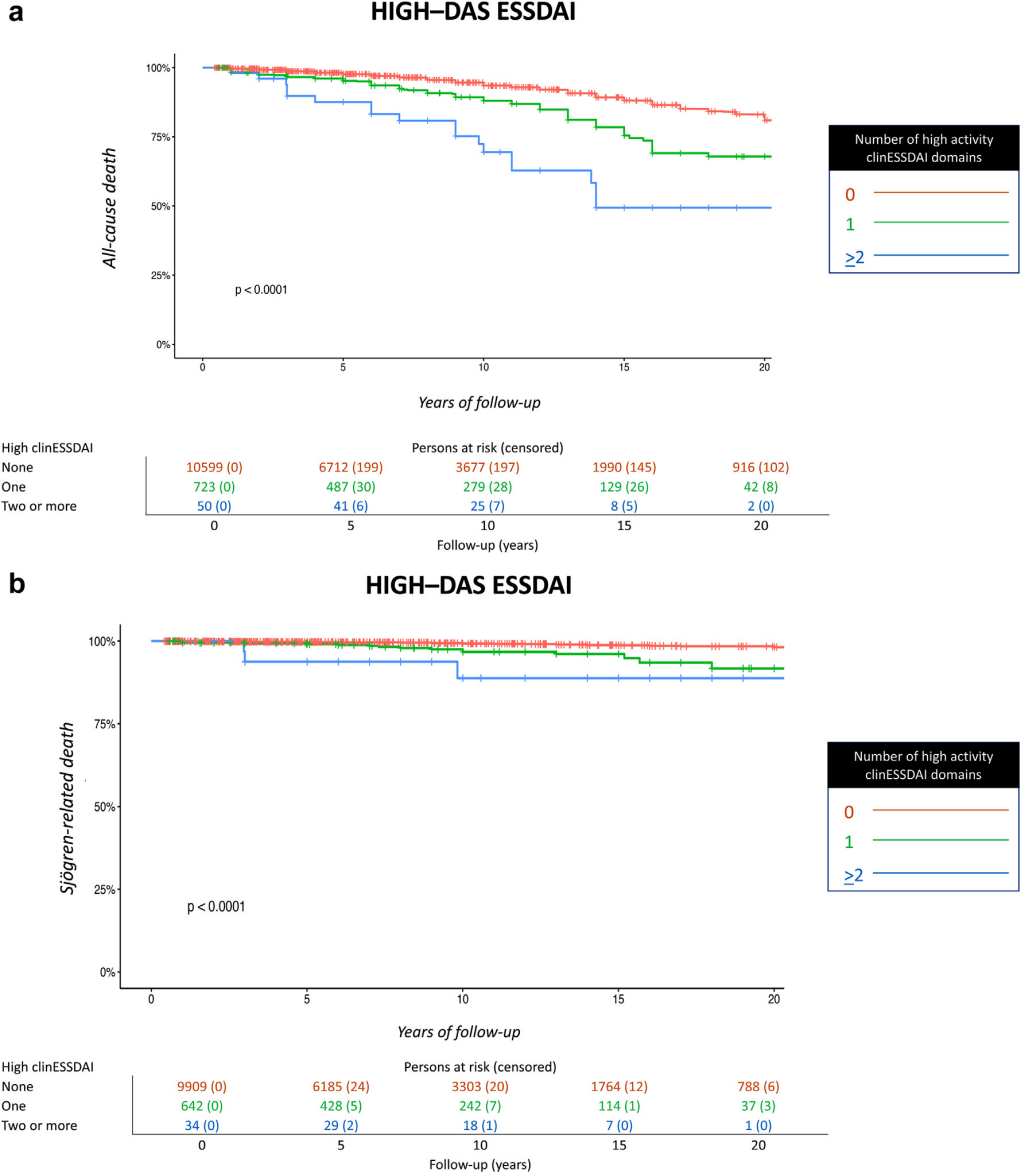
Cumulative survival for all-cause (**5a**) and SjS-related (**5b**) death according to the number of clinESSDAI domains classified as high activity at diagnosis (0, 1, 2 or more).

The sensitivity analysis stratifying systemic activity according to the DAS states showed that those patients presenting with a high DAS-ESSDAI at diagnosis had significantly worse survival compared to those with moderate or low DAS-ESSDAI (Fig. 4). The stratification of systemic activity according to the number of clinESSDAI domains classified as high activity at diagnosis yielded a non-adjusted HR for all-cause death of 1.89 (95% CI, 1.53–2.34) in those with one high activity domain and of 4.76 (95% CI, 3.02–7.50) for those with two or more high activity domains, with people without any high activity domain as the reference population. The non-adjusted HR for Sjögren-related death was 3.38 (95% CI, 1.95–5.83) for individuals with one high activity domain and 10.50 (95% CI, 3.30–33.40) for those with two or more, respectively (Fig. 5, Supplementary Table S6).

Univariate and multivariate-adjusted analyses of prognostic factors for the leading causes of death unrelated to systemic Sjögren (infection, cardiovascular disease, and solid malignancies) are summarized in Supplementary Tables S7–S12.

## Discussion

We present the data on mortality in primary SJS in the largest study conducted to date, including nearly 12,000 individuals who were followed for an average of 9 years, with almost 900 deaths identified. These figures are 15–30 times higher than those reported in previous studies,^6^, ^7^, ^8^, ^9^, ^10^, ^11^, ^12^, ^13^ ensuring an appropriate sample size not only for investigating risk factors for all-cause mortality but also for allowing us to investigate risk factors for Sjögren-related deaths specifically. We found that mortality in patients with SjS can be related to different aetiologies, often unrelated to the systemic autoimmune damage caused by the disease, as occurs with other diseases such as SLE or RA (29, 30). Only 14% of deaths in which the aetiology was detailed were directly related to systemic Sjögren (including haematological malignancies), while nearly 60% were related to infections and cardiovascular disease. A possible explanation for the low percentage of deaths directly attributed to SjS might be the low frequency of end-stage organ failure caused by systemic Sjögren and the excellent prognosis of the most frequent haematological malignancy reported in SjS patients (low-grade MALT lymphoma).^25^

We found that epidemiological profile and disease-related phenotype at diagnosis modulate the survival of people affected by the disease. The epidemiological profile played a significant role in the adjusted multivariate models, driven mainly by the age at which the disease is diagnosed; these are expected findings considering not only the influence of aging on the risk of death but also their recognized influence on the clinical and immunological phenotype of SjS.^26^^,^^27^ In contrast, the role of sex as a covariate in modulating mortality was less significant than expected based on previous studies,^6^^,^^13^ with men with SjS showing poor survival in only two out of the five multivariate models (Supplementary Table S13).

Among disease-related determinants of mortality, systemic activity scored according to the ESSDAI and cryoglobulinaemia were the two key baseline predictors of all-cause and Sjögren-related death. Firstly, high systemic activity at diagnosis (more than 14 points according to the DAS-ESSDAI classification) was associated with a 1.7-fold increased risk of all-cause death and a 1.6-fold increased risk of SjS-related death in the competing risk multivariate analysis. These results confirm a previous study conducted in 1045 Spanish patients, which first linked systemic activity defined according to the ESSDAI with systemic-cause mortality in SjS.^6^ We also found that severe multiorgan involvement (defined according to the number of clinESSDAI domains scored as high activity level at diagnosis) was an additional key mortality risk factor. The non-adjusted risk for all-cause death was 2.5-fold higher in individuals with two or more high activity domains (HR of 4.76) than those with only one high activity domain (HR of 1.89). Some previous studies have focused on the potentially life-threatening presentation of systemic SjS. Baldini et al.^28^ found severe systemic manifestations in 15% of patients, especially those with an immunological profile suggestive of B cell hyperactivation, while Flores-Chavez et al.^29^ identified 208 (13%) out of 1580 Spanish patients having an activity level scored as high in at least one ESSDAI domain, with an unadjusted, overall mortality rate of 20% that reached 33% in those individuals presenting two or more high ESSDAI domain involvements. Several studies have linked higher systemic activity scores at disease diagnosis with poor outcomes during the follow-up, overwhelmingly with the development of lymphoma^25^^,^^30^, ^31^, ^32^; only one confirmed the association between systemic activity (measured according to the ESSDAI) and reduced survival.^6^ Although it was not the objective of this study, we found a significant association between high systemic activity at diagnosis and poor survival due to infections and cardiovascular disease. These findings merit subsequent specific studies focused on these causes of death unrelated to SjS, especially considering that previous studies have reported an increased risk of both infections^33^, ^34^, ^35^ and cardiovascular disease^36^, ^37^, ^38^, ^39^, ^40^ in individuals with SjS.

The key SjS-related mortality risk factor identified in all the multivariate CPH models was cryoglobulinaemia, which has been reported by several studies conducted in smaller European cohorts from Spain, Italy, Hungary, and Greece.^15^^,^^21^^,^^41^, ^42^, ^43^ We have confirmed for the first time that cryoglobulins were associated with mortality independently from high systemic activity in a multivariate-adjusted model. This is an interesting finding considering that cryoglobulinaemia often overlaps with some clinical ESSDAI domains (skin, renal, and PNS domains).^44^ In addition, cryoglobulins are also a risk factor for the development of B-cell lymphoma, which is included as high activity in the lymphadenopathy/lymphoma ESSDAI domain.^45^ Therefore, cryoglobulins may play a central role in reducing the survival of individuals affected by SjS through two key pathogenic ways: promoting the development of severe systemic manifestations of the disease in vital organs and increasing the risk of developing B-cell lymphoma.

From a practical view, it is also helpful to highlight what SjS-related features at diagnosis do not influence survival. Glandular involvement has no impact, not only considering dryness symptoms but also on diagnostic tests, including ocular tests and salivary gland biopsy, confirming the results of previous studies.^22^ The exception was the reduced survival of patients presenting with oral glandular dysfunction observed in two of the five multivariate-adjusted CPH models (Supplementary Table S13), confirming the results of a previous single-centre study in which patients with severe involvement in salivary scintigraphy at diagnosis had a lower survival.^46^ Having low or moderate systemic activity at the time of diagnosis did not worsen survival of SjS, with only severe systemic activity (high DAS-ESSDAI) being associated with an increased risk of death. Finally, there were no significant differences in survival between Ro-positive and Ro-negative patients and between those with or without hypocomplementemia, in contrast with previous studies that linked hypocomplementemia (especially for low C4 levels) with poor survival.^10^^,^^14^^,^^47^ Considering that low C4 levels are linked to cryoglobulinaemia in more than 40% of patients with SjS^41^ and the influence of geolocation and ethnicity on the frequency of both cryoglobulinaemia and hypocomplementaemia,^48^ the specific characteristics of our study (internationally-based, multi-ethnic cohort) and the size of people studied (15-30-times larger than previous studies) can explain the lack of association found in our study.

The study results can be interpreted after considering the potential limitations of their design and methodology. The predominant presence of European patients may limit the generalization of the results beyond this ethnicity. In addition, the participant centres are mainly tertiary university hospitals that are considered the referral centre in their corresponding cities (and, in most cases, in their countries), contributing to a potential selection bias favoring the collection of individuals with a more severe clinical phenotype. The retrospective design in a real-world scenario allowed for missing information in some diagnostic variables. This is a fact to be considered when the results obtained from multivariate-adjusted models are interpreted, especially for cryoglobulins tested only in half the patients' total cohort. Another source of heterogeneity may be the availability of some diagnostic tests in all participating centres and their significant influence on the multivariate models concerning missing values. Information about the use of immunosuppressive therapies at diagnosis was not collected for several reasons, including the lack of previous studies linking their use with mortality in adjusted models, the lower frequency of their use in comparison with other systemic autoimmune diseases,^49^ and their potential collinearity with systemic activity, which is the reason for prescribing these therapies in SjS individuals. Information about socioeconomic status and concomitant non-communicable diseases at the time of diagnosis is not included in the MBDS of the study (except for haematological malignancies), and the lack of adjustment for these variables should be acknowledged as a limitation of the study in all-cause mortality analyses. Caution should also be exercised when interpreting the results of the competing risk analysis of Sjögren-related death, considering the potential bias introduced by treating competing events as censored and not accounting for the informative nature of these events.

The key methodological strength of this study is its large sample size, with nearly 12,000 patients (including about 6000 individuals tested for cryoglobulins) and the broadest international representation (27 countries from 5 continents). Previous studies have analysed SjS populations of less than 1000 patients, and most mortality rates are below 10% (the limit of what is considered the appropriate size when a sample from a total population is studied). Therefore, the sample size of the dependent variable (people who died) was too low, ranging between 30 and 50, affecting the reliability of their results. We consider that the results obtained after analysing a cohort of nearly 12,000 patients will be more representative of the worldwide SjS population.

In conclusion, the key mortality risk factors at the time of SjS diagnosis were positive cryoglobulins and a high systemic activity scored using the ESSDAI, conferring nearly two times increased risk of all-cause and Sjögren-related death. The risk increased to five times for all-cause death and up to ten times for Sjögren-systemic death in individuals presenting with severe multiorgan disease (at least two organs or systems involved and scored as highly active at diagnosis). Systemic involvement was directly involved in 14% of deaths in individuals with SjS, in more than half the cases due to haematological malignancies. These findings support the mandatory measurement of systemic activity and cryoglobulin testing at SjS diagnosis to anticipate better which patients will require closer follow-up. Further studies are needed to investigate the potential association between the SjS phenotype at diagnosis and the risk of dying due to causes unrelated to systemic disease, mainly cardiovascular events and infections.

## Contributors

All co-authors contributed to the manuscript (data collection, data interpretation, reading draft, approving manuscript). MRC did the statistical analysis. PBZ and AFC wrote the first draft of the report with input from MRC. MRC, PBZ & AFC has directly accessed to and verified the merged data. MRC was responsible for the decision to submit the manuscript.

## Data sharing statement

Data available on request due to privacy/ethical restrictions: The data that support the findings of this study are available on request from the corresponding author [MRC]. The data are not publicly available due to restrictions (information that could compromise the privacy of research participants).

## Declaration of interests

AR and ADF received the following Grant sor Contracts: R01 DE018209/DE/NIDCR NIH HHS/United States; U54 GM104938/GM/NIGMS NIH HHS/United States; P30 AR053483/AR/NIAMS NIH HHS/United States; P50 AR060804/AR/NIAMS NIH HHS/United States; R01 AR065953/AR/NIAMS NIH HHS/United States.

ADF received the following Grants: R01 AR074310/NIAMS NIH HHS/United States; Janssen Research and Development, LLC.

ADF reported the following patent: Antibody Tests for Identifying Ro Negative Sjögren's Syndrome and Use as Biomarkers for Dysregulated B Cell Responses, B Cell Lymphoma, Tissue Fibrosis and Salivary Gland Dysfunction. U.S. Patent application 17/797,619, filed August 4, 2022, European Patent application 21750408.3, filed September 14, 2022 and Canadian Patent application, filed September 14, 2022.

FA received Grants from Pfizer & Novartis, payments or honoraria from Abbvie, Pfizer, Galapagos, Novartis, BMS, Boeringher, Janssen, and participated on Safety/Advisory Boards of Janssen and Boeringher.

MR participated on Safety/Advisory Boards by Janssen and in clinical trials (BMS, Novartis, Servier).

PO participated on Safety/Advisory Boards by Fresenius Kabi, Novartis & Boehringer Ingelheim.

RG received Grants from Pfizer and Abbvie, payments/honoraria from Abbvie, Pfizer, MSD, Novartis, BMS and Boeringher, and participated on Safety/Advisory Boards by Abbvie, Pfizer, and Boeringher.

SR participated on Safety/Advisory Boards by Janssen.

TM declared that is working as medical advisor for UCB Pharma Sweden.

VCR declared that is working as medical advisor for UCB Pharma Sweden.

All other authors declare no competing interests.

## Appendix A Supplementary data

Supplementary Tables S1–S13

Consortium members names
