# Supplementary material for: Mortality risk factors in primary Sjögren syndrome: a real-world, retrospective, cohort study
Source: eClinicalMedicine. 2023 Jul 4;61:102062. doi: 10.1016/j.eclinm.2023.102062 (PMC10344811; doi:10.1016/j.eclinm.2023.102062)
Supplement: Supplementary Tables S1–S13 [file mmc1.pdf]

Supplementary Table 1. Predictive factors for all-cause death identified in the univariate CPH model in the entire cohort of patients (12802 patients, including 1430 with a follow-up of fewer than six months).

				Univariate model for all-cause death				
**Domains**	**Variables at diagnosis**	**Reference**	**Group at risk**	**Coefficient (B)**	**HR**	**95% CI (I)**	**95% CI (S)**	***P-value***
**Epidemiological**	Age	..	..	0.115	1.122	1.114	1.129	***<0.001***
	Sex	Women	Men	0.607	1.836	1.475	2.285	***<0.001***
	Ethnicity	White	Non-White	-0.237	0.789	0.641	0.972	***0.026***
	Healthcare score*	>80	<80	-0.309	0.735	0.627	0.860	***<0.001***
	History of HM	No	Yes	0.930	2.533	1.659	3.868	***<0.001***
**Glandular**	Dryness of mouth/eyes	Absence	Presence	-0.141	0.869	0.557	1.354	*0.534*
	Ocular tests	Normal	Abnormal	0.515	1.673	1.286	2.177	***<0.001***
	Oral tests	Normal	Abnormal	0.532	1.702	1.336	2.169	***<0.001***
	Salivary gland biopsy	Normal	FLS	-0.237	0.789	0.624	0.998	***0.048***
**Systemic**	clinESSDAI score	0	>=1	-0.042	0.959	0.839	1.096	*0.537*
	DAS state	No high	High	0.689	1.991	1.710	2.317	***<0.001***
**Immunological**	ANA	Negative	Positive	-0.133	0.876	0.739	1.037	*0.125*
	RF	Negative	Positive	-0.051	0.950	0.831	1.086	*0.454*
	Ro antibodies	Negative	Positive	-0.314	0.731	0.635	0.842	***<0.001***
	La antibodies	Negative	Positive	-0.159	0.853	0.749	0.972	***0.017***
	C3 levels	Normal	Low	-0.078	0.925	0.755	1.134	*0.454*
	C4 levels	Normal	Low	0.080	1.084	0.878	1.337	*0.455*
	Cryoglobulins	Negative	Positive	0.335	1.397	1.105	1.768	***0.005***

**WHO-UHC score country by country*

HM: hematological malignancy; ANA: antinuclear antibodies; RF: rheumatoid factor; FLS: focal lymphocytic sialoadenitis; DAS: disease activity state; HR: hazard ratio; CI: confidence interval

Supplementary Table 2. Predictive factors for all-cause death identified in the multivariate CPH model in the entire cohort of patients (12802 patients, including 1430 with a follow-up of fewer than six months). The multivariate model included all the epidemiological variables as covariates and those that achieved a p-value <0.1 in the univariate analysis of Supplementary Table 3 as independent variables.

				Multivariate adjusted model for all-cause death				
**Domains**	**Variables at diagnosis**	**Reference**	**Group at risk**	**Coefficient (B)**	**HR**	**95% CI (I)**	**95% CI (S)**	***P-value***
**Epidemiological**	Age	..	..	0.127	**1.136**	**1.120**	**1.152**	*<0.001*
	Sex	Women	Men	0.618	**1.854**	**1.170**	**2.940**	*0.009*
	Ethnicity	White	Non-White	0.344	1.411	0.685	2.904	*0.350*
	Healthcare score*	>80	<80	-0.113	0.893	0.652	1.224	*0.483*
	History of HM	No	Yes	-0.817	0.442	0.157	1.246	*0.123*
**Glandular**	Ocular tests	Normal	Abnormal	0.274	1.316	0.711	2.436	*0.382*
	Oral tests	Normal	Abnormal	0.663	**1.941**	**1.167**	**3.231**	*0.011*
	Salivary gland biopsy	Normal	FLS	-0.175	0.840	0.580	1.216	*0.354*
**Systemic**	DAS state	No high	High	0.532	**1.703**	**1.280**	**2.264**	*<0.001*
**Immunological**	Ro antibodies	Negative	Positive	-0.060	0.942	0.699	1.269	*0.693*
	La antibodies	Negative	Positive	-0.028	0.973	0.720	1.315	*0.858*
	Cryoglobulins	Negative	Positive	0.580	**1.786**	**1.252**	**2.548**	*0.001*

Supplementary Table 3. Specific causes of death in 640 patients with available information on the five leading subcategories (systemic SjS, cardiovascular events, infections, solid malignancy, and others).

	Number (n)
SYSTEMIC ACTIVITY	
Hematological neoplasia	54
Pulmonary fibrosis	23
Kidney renal failure	7
PAH-related pulmonary fibrosis	3
Systemic vasculitis	2
**TOTAL systemic activity**	**89**
INFECTION	
Pulmonary	83
Sepsis	31
Urinary	20
COVID-19	13
Other viruses	3
Abdominal	3
Not detailed	27
**TOTAL infection**	**180**
CARDIOVASCULAR	
Coronary/heart disease	115
Cerebral disease	45
Peripheral arterial disease	7
Not detailed	3
**TOTAL cardiovascular**	**170**
OTHERS	
Other chronic diseases	47
Accidental	4

Suicide	2
Not detailed	35
**TOTAL other causes**	**88**
SOLID MALIGNANCIES	
Lung	18
Breast	15
Colorectal	12
Pancreas	9
Gastric	5
Other	21
Not detailed	33
**TOTAL solid malignancies**	**113**

Supplementary Table 4. Predictive factors for SjS-related death identified in the univariate CPH model in the entire cohort of patients (12802 patients, including 1430 with a follow-up of fewer than six months).

				**Univariate model for Sjögren-related death**				
**Domains**	**Variables at diagnosis**	**Reference**	**Group at risk**	**Coefficient (B)**	**HR**	**95% CI (I)**	**95% CI (S)**	***P-value***
**Epidemiological**	Age	..	..	0.104	1.109	1.088	1.131	***<0.001***
	Sex	Women	Men	0.827	2.287	1.217	4.295	***0.010***
	Ethnicity	White	Non-White	-0.117	0.889	0.492	1.607	*0.698*
	Healthcare score*	>80	<80	-1.164	0.312	0.166	0.587	***<0.001***
	History of HM	No	Yes	2.257	9.554	4.621	19.750	***<0.001***
**Glandular**	Dryness of mouth/eyes	Absence	Presence	0.701	2.016	0.280	14.500	*0.486*
	Ocular tests	Normal	Abnormal	0.515	1.674	0.773	3.628	*0.192*
	Oral tests	Normal	Abnormal	0.917	2.502	1.085	5.768	***0.031***
	Salivary gland biopsy	Normal	FLS	-0.134	0.874	0.394	1.939	*0.741*
**Systemic**	clinESSDAI score	0	>=1	0.835	2.305	1.378	3.856	***0.001***
	DAS state	No high	High	1.394	4.030	2.658	6.111	***<0.001***
**Immunological**	ANA	Negative	Positive	1.035	2.815	1.231	6.437	***0.014***
	RF	Negative	Positive	-0.044	0.957	0.626	1.461	*0.838*
	Ro antibodies	Negative	Positive	-0.127	0.881	0.554	1.402	*0.593*
	La antibodies	Negative	Positive	-0.048	0.953	0.636	1.429	*0.817*
	C3 levels	Normal	Low	0.479	1.615	0.958	2.723	***0.072***
	C4 levels	Normal	Low	0.571	1.770	1.007	3.112	***0.047***
	Cryoglobulins	Negative	Positive	1.200	3.321	1.848	5.967	***<0.001***

**WHO-UHC score country by country*

HM: hematological malignancy; ANA: antinuclear antibodies; RF: rheumatoid factor; FLS: focal lymphocytic sialoadenitis; DAS: disease activity state; HR: hazard ratio; CI: confidence interval

Supplementary Table 5. Predictive factors for SjS-related death identified in the multivariate CPH model in the entire cohort of patients (12802 patients, including 1430 with a follow-up of fewer than six months). The multivariate model included all the epidemiological variables as covariates and those that achieved a p-value <0.1 in the univariate analysis of Supplementary Table 5 as independent variables.

				Multivariate model for Sjögren-related death				
**Domains**	**Variables at diagnosis**	**Reference**	**Group at risk**	**Coefficient (B)**	**HR**	**95% CI (I)**	**95% CI (S)**	*P-value*
**Epidemiological****	Age	..	..	0.141	**1.152**	**1.094**	**1.213**	*<0.001*
	Sex	Women	Men	-0.147	0.863	0.189	3.947	*0.850*
	Healthcare score*	>80	<80	-2.509	**0.081**	**0.010**	**0.640**	*<0.001*
**Glandular**	Oral tests	Normal	Abnormal	1.626	5.082	0.658	39.230	*0.119*
**Systemic**	clinESSDAI score	0	>=1	-0.349	0.705	0.249	2.001	*0.512*
	DAS state	No high	High	0.409	1.505	0.514	4.408	*0.456*
**Immunological**	ANA	Negative	Positive	0.251	1.285	0.282	5.852	*0.746*
	C3 levels	Normal	Low	0.816	2.262	0.767	6.676	*0.139*
	C4 levels	Normal	Low	0.424	1.527	0.447	5.217	*0.499*
	Cryoglobulins	Negative	Positive	1.461	**4.309**	**1.692**	**10.975**	*0.002*

**WHO-UHC score country by country*

HM: hematological malignancy; ANA: antinuclear antibodies; RF: rheumatoid factor; FLS: focal lymphocytic sialoadenitis; DAS: disease activity state; HR: hazard ratio; CI: confidence interval

***Ethnicity and history of HM excluded from the model due to low number of events*

Supplementary Table 6. HRs and 95% confidence interval for all-cause and SjS-related death according to the number of clinESSDAI domains classified as high activity at diagnosis (0, 1, 2 or more) yielded by a non-adjusted CPH model.

	Reference	***High clinESSDAI domains = 0***	
	Groups at risk	***High clinESSDAI domain = 1***	***High clinESSDAI domain => 2***
**All-cause death**	**HR**	**1.89**	**4.76**
	95% CI (I)	1.53	3.02
	95% CI (S)	2.34	7.50
**Sjögren-related death**	**HR**	**3.38**	**10.50**
	95% CI (I)	1.95	3.30
	95% CI (S)	5.83	33.40

Supplementary Table 7. Predictive factors for cardiovascular-related death (coronary/heart disease, cerebrovascular disease, peripheral artery disease/others) identified in the univariate CPH model.

Domains	Variables at diagnosis	Reference	Group at risk	Coefficient (B)	HR	95% CI (I)	95% CI (S)	P-value
EPIDEMIOLOGICAL	Mean age at diagnosis	..	..	0.114	1.121	1.105	1.138	<0.001
	Gender	Women	Men	0.797	2.218	1.392	3.534	0.001
	Ethnicity	White	Non-White	-0.474	0.622	0.428	0.905	0.013
GLANDULAR	Dryness of mouth and/or eyes	Absence	Presence	-0.602	0.548	0.242	1.240	0.149
	Ocular studies	Normal	Abnormal	0.681	1.977	1.072	3.645	0.029
	Oral studies	Normal	Abnormal	0.129	1.137	0.729	1.774	0.571
	Minor salivary gland biopsy	Normal	FLS	-0.042	0.959	0.512	1.796	0.802
SYSTEMIC	Systemic activity	clinESSDAI = 0	clinESSDAI>=1	0.802	2.231	1.542	3.229	<0.001
		Other DAS-clinESSDAI	High DAS-clinESSDAI	1.419	4.134	3.063	5.580	<0.001
IMMUNOLOGICAL	ANA	Negative	Positive	-0.101	0.904	0.613	1.332	0.609
	RF	Negative	Positive	0.058	1.060	0.786	1.429	0.704
	Ro	Negative	Positive	-0.256	0.774	0.560	1.071	0.122
	La	Negative	Positive	-0.097	0.908	0.677	1.218	0.519
	C3	Normal	Low levels	0.269	1.309	0.879	1.948	0.185
	C4	Normal	Low levels	0.445	1.560	1.037	2.348	0.033
	Cryoglobulins	Negative	Presence	0.281	1.325	0.802	2.189	0.272

ANA: antinuclear antibodies; RF: rheumatoid factor; FLS: focal lymphocytic sialoadenitis; DAS: disease activity state; HR: hazard ratio; CI: confidence interval

Supplementary Table 8. Predictive factors for cardiovascular-related death identified in the multivariate CPH model. The multivariate model included all the epidemiological variables as covariates and those that achieved a p-value <0.1 in the univariate analysis of Supplementary Table 8 as independent variables.

Domains	Variables at diagnosis	Reference	Group at risk	Multivariate model for infection-cause death Coefficient (B)	HR	95% CI (I)	95% CI (S)	P-value
EPIDEMIOLOGICAL	Mean age at diagnosis	..	..	0.122	1.129	1.111	1.148	<0,001
	Gender	Women	Men	0.579	1.785	1.058	3.010	0.030
	Ethnicity	White	Non-White	0.946	2.575	1.674	3.961	<0,001
GLANDULAR	Ocular studies	Normal	Abnormal	0.328	1.388	0.704	2.737	0.344
SYSTEMIC	Systemic activity	clinESSDAI = 0	clinESSDAI>=1	0.608	1.836	1.158	2.911	0.010
		Other DAS-clinESSDAI	High DAS-clinESSDAI	1.294	3.647	2.563	5.189	<0,001
IMMUNOLOGICAL	C4	Normal	Low levels	0.192	1.212	0.786	1.868	0.384

DAS: disease activity state; HR: hazard ratio; CI: confidence interval

Supplementary Table 9. Predictive factors for infectious-related death identified in the univariate CPH model.

Domains	Variables at diagnosis	**Reference**	**Group at risk**	**Coefficient (B)**	**HR**	**95% CI (I)**	**95% CI (S)**	**P-value**
EPIDEMIOLOGICAL	Mean age at diagnosis	..	..	0.182	1.199	1.178	1.221	<0,001
	Gender	Women	Men	-0.095	0.909	0.447	1.850	0.792
	Ethnicity	White	Non-White	0.427	1.532	0.897	2.617	0.118
GLANDULAR	Dryness of mouth and/or eyes	Absence	Presence	-0.062	0.940	0.299	2.952	0.915
	Ocular studies	Normal	Abnormal	1.400	4.053	1.658	9.912	0.002
	Oral studies	Normal	Abnormal	0.775	2.170	1.170	4.024	0.014
	Minor salivary gland biopsy	Normal	FLS	-0.589	0.555	0.334	0.924	0.023
SYSTEMIC	Systemic activity	clinESSDAI = 0	clinESSDAI>=1	0.541	1.718	1.208	2.444	0.003
		Other DAS-clinESSDAI	High DAS-clinESSDAI	0.812	2.252	1.591	3.189	<0,001
IMMUNOLOGICAL	ANA	Negative	Positive	-0.180	0.836	0.562	1.243	0.375
	RF	Negative	Positive	0.375	0.864	0.636	1.174	0.350
	Ro	Negative	Positive	-0.061	0.941	0.661	1.339	0.735
	La	Negative	Positive	-0.061	0.941	0.694	1.276	0.695
	C3	Normal	Low levels	-0.218	0.804	0.495	1.304	0.377
	C4	Normal	Low levels	-0.235	0.790	0.462	1.351	0.390
	Cryoglobulins	Negative	Presence	-0.317	0.729	0.390	1.362	0.321

ANA: antinuclear antibodies; RF: rheumatoid factor; FLS: focal lymphocytic sialoadenitis; DAS: disease activity state; HR: hazard ratio; CI: confidence interval

Supplementary Table 10. Predictive factors for infectious-related death identified in the multivariate CPH model. The multivariate model included all the epidemiological variables as covariates and those that achieved a p-value <0.1 in the univariate analysis of Supplementary Table 10 as independent variables.

Domains	Variables at diagnosis	**Reference**	**Group at risk**	**Coefficient (B)**	**HR**	**95% CI (I)**	**95% CI (S)**	**P-value**
EPIDEMIOLOGICAL	Mean age at diagnosis	..	..	0.183	1.201	1.169	1.233	<0,001
	Gender	Women	Men	-0.670	0.512	0.125	2.093	0.351
	Ethnicity	White	Non-White	0.138	1.148	0.458	2.882	0.768
GLANDULAR	Ocular studies	Normal	Abnormal	0.900	2.460	0.770	7.855	0.129
	Oral studies	Normal	Abnormal	0.622	1.863	0.737	4.711	0.188
	Minor salivary gland biopsy	Normal	FLS	-0.502	0.605	0.355	1.033	0.066
SYSTEMIC	Systemic activity	clinESSDAI = 0	clinESSDAI>=1	0.463	1.589	0.946	2.668	0.080
		Other DAS-clinESSDAI	High DAS-clinESSDAI	0.535	1.707	1.013	2.878	0.045

DAS: disease activity state; HR: hazard ratio; CI: confidence interval

Supplementary Table 11. Predictive factors for solid malignancy-related death identified in the univariate CPH model.

Domains	Variables at diagnosis	**Reference**	**Group at risk**	**Coefficient (B)**	**HR**	**95% CI (I)**	**95% CI (S)**	**P-value**
EPIDEMIOLOGICAL	Mean age at diagnosis	..	..	0.104	1.110	1.089	1.130	<0.001
	Gender	Women	Men	0.295	1.343	0.654	2.759	0.421
	Ethnicity	White	Non-White	0.751	2.119	1.029	4.363	0.042
GLANDULAR	Dryness of mouth and/or eyes	Absence	Presence	-0.335	0.716	0.227	2.260	0.568
	Ocular studies	Normal	Abnormal	0.143	1.154	0.599	2.225	0.669
	Oral studies	Normal	Abnormal	0.432	1.541	0.795	2.985	0.200
	Minor salivary gland biopsy	Normal	FLS	-0.497	0.608	0.326	1.133	0.117
SYSTEMIC	Systemic activity	clinESSDAI = 0	clinESSDAI>=1	-0.371	0.690	0.476	1.001	0.051
		Other DAS-clinESSDAI	High DAS-clinESSDAI	0.224	1.251	0.755	2.073	0.385
IMMUNOLOGICAL	ANA	Negative	Positive	-0.250	0.779	0.488	1.243	0.295
	RF	Negative	Positive	-0.032	0.969	0.655	1.432	0.873
	Ro	Negative	Positive	0.002	1.002	0.646	1.554	0.992
	La	Negative	Positive	-0.314	0.730	0.500	1.066	0.104
	C3	Normal	Low levels	-0.856	0.425	0.185	0.975	0.043
	C4	Normal	Low levels	-0.284	0.753	0.364	1.559	0.445
	Cryoglobulins	Negative	Presence	0.506	1.659	0.839	3.281	0.146

ANA: antinuclear antibodies; RF: rheumatoid factor; FLS: focal lymphocytic sialoadenitis; DAS: disease activity state; HR: hazard ratio; CI: confidence interval

Supplementary Table 12. Predictive factors for solid malignancy-related death identified in the multivariate CPH model. The multivariate model included all the epidemiological variables as covariates and those that achieved a p-value <0.1 in the univariate analysis of Supplementary Table 12 as independent variables.

				**Multivariate model for solid malignancy-cause death**				
Domains	Variables at diagnosis	**Reference**	**Group at risk**	**Coefficient (B)**	**HR**	**95% CI (I)**	**95% CI (S)**	**P-value**
EPIDEMIOLOGICAL	Mean age at diagnosis	NA	NA	0.101	1.106	1.083	1.129	<0.001
	Ethnicity	White	Non-White	0.383	1.466	0.674	3.192	0.335
SYSTEMIC	Systemic activity	clinESSDAI = 0	clinESSDAI>=1	-0.185	0.831	0.540	1.279	0.400
IMMUNOLOGICAL	C3	Normal	Low levels	-0.597	0.551	0.237	1.277	0.165

HR: hazard ratio; CI: confidence interval

Supplementary Table 13. Summary of results obtained for the covariates and independent variables in the five statistical models. In blue, p values <0.05 in univariate analysis. In red, HR in the multivariate-adjusted CPH models.

Domains	Variables at diagnosis	Reference	Group at risk	**All-cause**	**All-cause 12K**	**Sjo-related**	**Sjo-competing**	**Sjo-related 12K**
**EPIDEMIOLOGICAL**	Age	..	..	1.13	1.14	1.14	1.12	1.15
	Sex	Women	Men		1.85		1.77	
	Ethnicity	White	Non-White					
	Healthcare score*	>80	<80					0.07
	History of HM	No	Yes					
**GLANDULAR**	Dryness of mouth/eyes	Absence	Presence					
	Ocular tests	Normal	Abnormal					
	Oral tests	Normal	Abnormal		1.94		1.38	
	Salivary gland biopsy	Normal	FLS					
**SYSTEMIC**	clinESSDAI score	0	>=1					
	DAS state	No high	High	1.68	1.7		1.55	
**IMMUNOLOGICAL**	ANA	Negative	Positive					
	RF	Negative	Positive					
	Ro antibodies	Negative	Positive					
	La antibodies	Negative	Positive					
	C3 levels	Normal	Low					
	C4 levels	Normal	Low					
	Cryoglobulins	Negative	Positive	1.72	1.79	2.57	1.52	1.71

Univ p<0.1
Multiv

**WHO-UHC score country by country*

HM: hematological malignancy; ANA: antinuclear antibodies; RF: rheumatoid factor; FLS: focal lymphocytic sialoadenitis; DAS: disease activity state; HR: hazard ratio; CI: confidence interval

Appendix. Other members of the Sjögren Big Data Consortium-Sjögren GEAS-SEMI who contributed to this study

Initial	Family name	Center
S	Arends	Department of Rheumatology & Clinical Immunology, University of Groningen, University Medical Center Groningen, Groningen, The Netherlands
E	Treppo	Clinic of Rheumatology, Department of Medical and Biological Sciences, University Hospital "Santa Maria della Misericordia", Udine, Italy
S	Longhino	Clinic of Rheumatology, Department of Medical and Biological Sciences, University Hospital "Santa Maria della Misericordia", Udine, Italy
V	Manfrè	Clinic of Rheumatology, Department of Medical and Biological Sciences, University Hospital "Santa Maria della Misericordia", Udine, Italy
M	Rizzo	Clinic of Rheumatology, Department of Medical and Biological Sciences, University Hospital "Santa Maria della Misericordia", Udine, Italy
C	Baldini	Rheumatology Unit, University of Pisa, Pisa, Italy
S	Bombardieri	Rheumatology Unit, University of Pisa, Pisa, Italy
M	Bandeira	Rheumatology Department, Hospital de Santa Maria, Centro Hospitalar Universitário Lisboa Norte and Rheumatology Research Unit, Instituto de Medicina Molecular, Faculdade de Medicina, Universidade de Lisboa, Lisbon Academic Medical Centre, Lisbon, Portugal
M	Silvéiro-António	Rheumatology Department, Hospital de Santa Maria, Centro Hospitalar Universitário Lisboa Norte and Rheumatology Research Unit, Instituto de Medicina Molecular, Faculdade de Medicina, Universidade de Lisboa, Lisbon Academic Medical Centre, Lisbon, Portugal
R	Seror	Center fo Immunology of Viral Infections and Autoimmune Diseases, Assistance Publique – Hôpitaux de Paris, Hôpitaux Universitaires Paris-Sud, Le Kremlin-Bicêtre, Université Paris Sud, INSERM, Paris, France Paris, France
X	Mariette	Center fo Immunology of Viral Infections and Autoimmune Diseases, Assistance Publique – Hôpitaux de Paris, Hôpitaux Universitaires Paris-Sud, Le Kremlin-Bicêtre, Université Paris Sud, INSERM, Paris, France Paris, France
G	Nordmark	Rheumatology, Department of Medical Sciences, Uppsala University, Uppsala, Sweden
D	Danda	Department of Clinical Immunology & Rheumatology, Christian Medical College & Hospital, Vellore, India
P	Wiland	Department of Rheumatology and Internal Medicine, Wroclaw Medical University, Wroclaw, Poland
R	Gerli	Rheumatology Unit, Department of Medicine, University of Perugia, Italy

SK	Kwok	Division of Rheumatology, Department of Internal Medicine, Seoul St. Mary's Hospital, College of Medicine, The Catholic University of Korea, Seoul, South Korea
SH	Park	Division of Rheumatology, Department of Internal Medicine, Seoul St. Mary's Hospital, College of Medicine, The Catholic University of Korea, Seoul, South Korea
M	Kvarnstrom	Department of Medicine, Solna, Division of Experimental Rheumatology, Karolinska Institutet, and Karolinska University Hospital, Stockholm
M	Wahren-Herlenius	Department of Medicine, Solna, Division of Experimental Rheumatology, Karolinska Institutet, and Karolinska University Hospital, Stockholm
S	Downie-Doyle	Department of Rheumatology, The Queen Elizabeth Hospital, University of Adelaide, South Australia, Australia
D	Sene	Service de Médecine Interne 2, Hôpital Lariboisière, Université Paris VII, Assistance Publique-Hôpitaux de Paris, 2, Paris, France
D	Isenberg	Centre for Rheumatology, Division of Medicine , University College London , UK
V	Valim	Department of Medicine, Federal University of Espírito Santo, Vitória, Brazil
V	Devauchelle-Pensec	Rheumatology Department, Brest University, INSERM 1227, Brest, France
A	Saraux	Rheumatology Department, Brest University, INSERM 1227, Brest, France
J	Morel	Department of Rheumatology, Teaching hospital and University of Montpellier, Montpellier, France
C	Morcillo	Autoimmune Diseases Unit, Research and Innovation Group in Autoimmune Diseases, Sanitas Digital Hospital, Hospital-CIMA-Centre Mèdic Milenium Balmes Sanitas, Barcelona, Spain
PE	Díaz Cuiza	Departamento de Reumatología del Seguro Social Universitario y consultorio privado de Reumatología, Sucre-Bolivia
BE	Herrera	Departamento de Reumatología del Seguro Social Universitario y consultorio privado de Reumatología, Sucre-Bolivia
L	González-de-Paz	Primary Healthcare Transversal Research Group, Primary Care Center Les Corts, CAPSBE, Barcelona, Spain
A	Sisó-Almirall	Primary Healthcare Transversal Research Group, Primary Care Center Les Corts, CAPSBE, Barcelona, Spain
