# Supplementary material for: Mortality risk factors in primary Sjögren syndrome: a real-world, retrospective, cohort study
Source: eClinicalMedicine. 2023 Jul 4;61:102062. doi: 10.1016/j.eclinm.2023.102062 (PMC10344811; doi:10.1016/j.eclinm.2023.102062)
Supplement: Consortium members names [file mmc2.docx]

| Initial | Family name | Center |
| --- | --- | --- |
| S | Arends | Department of Rheumatology & Clinical Immunology, University of Groningen, University Medical Center Groningen, Groningen, The Netherlands |
| E | Treppo | Clinic of Rheumatology, Department of Medical and Biological Sciences, University Hospital "Santa Maria della Misericordia", Udine, Italy |
| S | Longhino | Clinic of Rheumatology, Department of Medical and Biological Sciences, University Hospital "Santa Maria della Misericordia", Udine, Italy |
| V | Manfrè | Clinic of Rheumatology, Department of Medical and Biological Sciences, University Hospital "Santa Maria della Misericordia", Udine, Italy |
| M | Rizzo | Clinic of Rheumatology, Department of Medical and Biological Sciences, University Hospital "Santa Maria della Misericordia", Udine, Italy |
| C | Baldini | Rheumatology Unit, University of Pisa, Pisa, Italy |
| S | Bombardieri | Rheumatology Unit, University of Pisa, Pisa, Italy |
| M | Bandeira | Rheumatology Department, Hospital de Santa Maria, Centro Hospitalar Universitário Lisboa Norte and Rheumatology Research Unit, Instituto de Medicina Molecular, Faculdade de Medicina, Universidade de Lisboa, Lisbon Academic Medical Centre, Lisbon, Portugal |
| M | Silvéiro-António | Rheumatology Department, Hospital de Santa Maria, Centro Hospitalar Universitário Lisboa Norte and Rheumatology Research Unit, Instituto de Medicina Molecular, Faculdade de Medicina, Universidade de Lisboa, Lisbon Academic Medical Centre, Lisbon, Portugal |
| R | Seror | Center fo Immunology of Viral Infections and Autoimmune Diseases, Assistance Publique – Hôpitaux de Paris, Hôpitaux Universitaires Paris-Sud, Le Kremlin-Bicêtre, Université Paris Sud, INSERM, Paris, France Paris, France |
| X | Mariette | Center fo Immunology of Viral Infections and Autoimmune Diseases, Assistance Publique – Hôpitaux de Paris, Hôpitaux Universitaires Paris-Sud, Le Kremlin-Bicêtre, Université Paris Sud, INSERM, Paris, France Paris, France |
| G | Nordmark | Rheumatology, Department of Medical Sciences, Uppsala University, Uppsala, Sweden |
| D | Danda | Department of Clinical Immunology & Rheumatology, Christian Medical College & Hospital, Vellore, India |
| P | Wiland | Department of Rheumatology and Internal Medicine, Wroclaw Medical University, Wroclaw, Poland |
| R | Gerli | Rheumatology Unit, Department of Medicine, University of Perugia, Italy |
| SK | Kwok | Division of Rheumatology, Department of Internal Medicine, Seoul St. Mary's Hospital, College of Medicine, The Catholic University of Korea, Seoul, South Korea |
| SH | Park | Division of Rheumatology, Department of Internal Medicine, Seoul St. Mary's Hospital, College of Medicine, The Catholic University of Korea, Seoul, South Korea |
| M | Kvarnstrom | Department of Medicine, Solna, Division of Experimental Rheumatology, Karolinska Institutet, and Karolinska University Hospital, Stockholm |
| M | Wahren-Herlenius | Department of Medicine, Solna, Division of Experimental Rheumatology, Karolinska Institutet, and Karolinska University Hospital, Stockholm |
| S | Downie-Doyle | Department of Rheumatology, The Queen Elizabeth Hospital, University of Adelaide, South Australia, Australia |
| D | Sene | Service de Médecine Interne 2, Hôpital Lariboisière, Université Paris VII, Assistance Publique-Hôpitaux de Paris, 2, Paris, France |
| D | Isenberg | Centre for Rheumatology, Division of Medicine , University College London , UK |
| V | Valim | Department of Medicine, Federal University of Espírito Santo, Vitória, Brazil |
| V | Devauchelle-Pensec | Rheumatology Department, Brest University, INSERM 1227, Brest, France |
| A | Saraux | Rheumatology Department, Brest University, INSERM 1227, Brest, France |
| J | Morel | Department of Rheumatology, Teaching hospital and University of Montpellier, Montpellier, France |
| C | Morcillo | Autoimmune Diseases Unit, Research and Innovation Group in Autoimmune Diseases, Sanitas Digital Hospital, Hospital-CIMA-Centre Mèdic Milenium Balmes Sanitas, Barcelona, Spain |
| PE | Díaz Cuiza | Departamento de Reumatología del Seguro Social Universitario y consultorio privado de Reumatología, Sucre-Bolivia |
| BE | Herrera | Departamento de Reumatología del Seguro Social Universitario y consultorio privado de Reumatología, Sucre-Bolivia |
| L | González-de-Paz | Primary Healthcare Transversal Research Group, Primary Care Center Les Corts, CAPSBE, Barcelona, Spain |
| A | Sisó-Almirall | Primary Healthcare Transversal Research Group, Primary Care Center Les Corts, CAPSBE, Barcelona, Spain |
